# Pregnant women’s well-being and worry during the COVID-19 pandemic: a cross-sectional study

**DOI:** 10.1186/s12884-021-03548-4

**Published:** 2021-01-15

**Authors:** Forough Mortazavi, Maryam Mehrabadi, Roya KiaeeTabar

**Affiliations:** 1 Non-Communicable Diseases Research Center, Sabzevar University of Medical Sciences, Pardis Building, Towhid Blvd, Sabzevar, Khorasan Razavi Iran; 2 Health Chancellery, Sabzevar University of Medical Sciences, Sabzevar, Iran; 3 Mobini hospital, Sabzevar University of Medical Sciences, Sabzevar, Iran

**Keywords:** Well-being, Anxiety, Fear, Worry, Women, Pregnancy, COVID-19

## Abstract

**Background:**

COVID-19 caused some worries among pregnant women. Worries during pregnancy can affect women’s well-being. We investigated worry and well-being and associated factors among pregnant women during the COVID-19 pandemic.

**Methods:**

This descriptive cross-sectional study was conducted on 484 pregnant women using an online questionnaire. Sampling was performed in a period between May 5 and Aug 5, 2020. Inclusion criteria were having a single healthy fetus and having no significant psychological disorder. We collected the data using the Persian versions of the World Health Organization’s Well-Being Index (WHO-5 Well-Being Index) and the Cambridge Worry Scale. We used univariate and multivariate logistic regression analyses to identify predictors of women’s worry and well-being.

**Results:**

The mean total scores of the WHO-5 Well-Being Index and the percentage of WHO-5 score < 50 were 64.9 ± 29.0 and 24.4%, respectively. Predictors of women’s worry are the increased level of fear of COVID-19 (OR = 6.40, *p* <  0.001), a low family income (OR = 3.41, *p* <  0.001), employment status (OR = 1.86, *p* = 0.019), nulliparity (OR = 1.68, *p* = 0.024), having a COVID-19 infected person among relatives (OR = 2.45, *p* = 0.036), having a history of abortion (OR = 1.86, *p* = 0.012), having participated in the study after the first wave of COVID-19 outbreak (OR = 2.328, *p* = 0.003), and women’s age < 30 year (OR = 2.11, *p* = 0.002). Predictors of low level of well-being in pregnant women are worry about their own health and relationships (OR = 1.789, *p* = .017), worry about fetus health (OR = 1.946, *p* = 0.009), and having at least one infected person with COVID-19 among relatives (OR = 2.135, *p* = 0.036).

**Conclusions:**

The percentage of women experiencing a low well-being state was relatively high. This result is worthy of attention by health care providers and policy makers. Providing care and support to pregnant women should have high priority during the COVID-19 pandemic.

**Supplementary Information:**

The online version contains supplementary material available at 10.1186/s12884-021-03548-4.

## Background

Pregnancy has been described as one of the most pleasant and critical periods in the most women’s lives which involves new emotions and experiences. Unfortunately, since the COVID-19 pandemic, pregnancy and childbirth for women are taking place in difficult conditions. Several issues have contributed to increasing concerns among people and in particular in pregnant women including the stressful news of the number of infected individuals and the death toll, the diverse symptoms and complications caused by the disease, and our limited knowledge about the disease [1].

Pregnant women have been severely affected by previous epidemics [2]; so, some researchers predicted that COVID-19 might have similar impact on pregnant women. So far, many questions relating to COVID-19 in pregnant women remain without clear and satisfactory answers. These include questions about the possibility of women-to-child transmission, the effect of COVID-19 on the fetus, and the adverse effects of the disease on pregnant women [2].

The level of anxiety and fear among pregnant women increased during the COVID-19 pandemic [3, 4]. This is attributable to a number of factors including the probability of the increased risk of getting COVID-19 or presenting severe complications, the risk of death in infected pregnant women, and the uncertainty about the effectiveness of the available treatments and timely vaccine production [5]. Under normal circumstances, 80% of women’s main concerns about pregnancy and childbirth are nothing extraordinary and unusual. Only about 20% of pregnant women experience excessive concern about future events in pregnancy [6]. A study on Indian obstetricians showed that during COVID-19 pandemic the level of fear and worry were increased in pregnant women [4].

Fear, worry, and anxiety during pregnancy has negative physical and psychological health consequences for pregnant women. Previous studies suggest that pregnancy stress may lead to mother-infant relationship disorder, antenatal and postpartum depression, increased physical problems [7, 8], and an increased risk of pre-eclampsia [9]. Rashidi and her colleague have reported an increased preference for cesarean during the COVID-19 pandemic in Tehran [10].

In addition to worry about COVID-19, pregnancy brings specific worries to women. COVID-19 related worries may elevate some pregnancy specific worries such as worry about fetus health or mother’s own health and worry about going to hospital. Several studies have attempted to understand the nature of worries in pregnant women and have developed scales to measure the nature and the extent of pregnant women’s worries. Results of these studies indicate that pregnant women’s worries originate from different sources. These sources can be classified as socio-medical, socio-economic, the health of the fetus, mother’s own health and relational issues [11, 12].

Well-being is considered to consist of two components: feeling healthy and relatively robust and being able to carry out ones job and other tasks satisfactorily. There are two sides to wellbeing: a psychological and an emotional one [13]. Psychological well-being involve reflexive consideration and appraisals of one’s own life. Emotional well-being is more a matter experiencing positive and life enhancing emotions such as security, joy, and contentment [14]. The World Health Organization-5 Well-Being Index (WHO-5 Well-Being Index) has been developed to screen for depressive symptoms. It has been also used to monitor emotional well-being and psychological well-being [15].

With regard to the stress and concerns of pregnant women that were reported above, we wanted to explore the extent that these concerns and fears had negative effect on maternal well-being. In addition, we aimed to investigate well-being of pregnant women and its associated factors during the COVID-19 pandemic.

## Methods

We conducted this descriptive cross-sectional study on pregnant women who were registered in health centers affiliated to Sabzevar University of Medical Sciences during the COVID-19 pandemic. We collected the data using an online questionnaire. Midwives working in health centers affiliated to Sabzevar University of Medical Sciences used text messages to invite 693 pregnant women registered with them to participate in the study. Overall, 484 women responded to the invitation and participated in the study (response rate = 69.8%). Those who responded to the invitation received an informed consent form, and then were provided with the link for the online questionnaire. Sampling was performed between May 5 and Aug 5, 2020. Inclusion criteria were being pregnant at the time of the study, having a single healthy fetus, informed consent to participate in the study, and having no significant psychological disorder. To determine the sample size, we considered the percentage of women in a previous study whose worry score was less than 37 (the median score of the worry scale) [12] and also the percentage of women in another study whose well-being score was less than 50 [16]. These percentages were 45 and 34.5%, respectively. We calculated the sample size using Cochran’s formula (pqz^2^/d^2^) with the confidence level of 95% and a margin of error of 5%. The minimum sample size estimate is 347. We collected the data using the Persian versions of the WHO-5 Well-Being Index [16] and the Cambridge Worry Scale (CWS) [12]. In addition, we developed a questionnaire to investigate socio-demographic and obstetric information (supplementary file).

### Instruments

We collected the data on maternal socio-demographic and obstetric information (such as age, level of education, employment status, monthly family income, gestational age, parity, having a chronic disease or pregnancy complication, having at least one COVID-19 infected person in their relatives, and having at least one death due to COVID-19 in relatives). To measure the fear of COVID-19, we designed one question with a likert-type scale ranging from “not at all” (1) to “severe” (5) and asked women to rate their fear (supplementary file).

#### Cambridge worry scale (CWS)

Green and colleagues developed the Cambridge Worry Scale (CWS) with 17 items [11] to measure worry content among individuals. Scores are ranged from “not a worry” (0) to “major worry” (5). The total score of the CWS ranges from zero to 85, with a higher score representing the severity of worries. The CWS includes four subscales as follow: socio-medical, own health, socio-economic, and relational. The reliability of the CWS was aceptable (Cronbach’s alpha = 0.76). We translated the scale into Persian and confirmed its validity among pregnant women. The Persian version consists of 23 items and 4 factors including the socio medical (10 item)(such as “Having nobody in delivery ward”, “Giving birth”, “Whether midwives provide good care in labor”, “Going to hospital”, “Crowded delivery ward”), health of mother/other & Relationships (4 items)(i.e., “Your own health”, “The health of someone closes to you”, “Your relationship with your husband”, “Your relationship with your family and friends”), fetus health (3 items)(i.e., “The possibility of miscarriage”, “The possibility of fetal death, disease or anomaly”, “The probability of going into labor too early”) and socio-demographic factor (6 items) (such as “Money problems”, “Employment problems”). The total score of the Persian CWS ranges from zero to 115 with the median score of 37. The Cronbach’s alpha value for the 23 items of the Persian CWS was 0.886 [12]. We used the Persian version of the CWS for investigating pregnant women’s worries during the COVID-19 pandemic because we believe its items are relevant to the pregnant women’s worries during the pandemic.

#### The World Health Organization’s well-being index (WHO-5 well-being index)

The World Health Organization’s well-being Index (*WHO-5 Well-Being Index*) was designed to assess the well-being of individuals over the past 2 weeks [17]. It consists of five positively worded items with a 6-point Likert scale (such as “I have felt cheerful and in good spirits”). Each item is rated from zero to five. Zero indicates experiencing good feelings at no time and five indicates experiencing good feelings all the time. The minimum and maximum score of the scale are 0 and 25 respectively. The total score is usually transformed to a scale of 0 to 100. The cut-off point of 50 was considered for screening for depression. Individuals with scores less than fifty are referred for further evaluations. The World Health Organization translated the scale into several languages including Persian [17]. Mortazavi and Colleagues investigated the validity and reliability of the Persian version in pregnant women and the results indicates that the scale is unidimentionl with excellent reliability (Cronbach’s alpha = 0.85) [16].

### Data analysis

We analyzed the data using SPSS software (SPSS Inc. Released 2009. PASW Statistics for Windows, Version 18.0. Chicago: SPSS Inc.). We used chi-square test of independence to investigate the relationships between categorical variables. We used t-test to compare the mean scores of the worry factors classified according to the levels of the WHO-5 Well-Being Index.

We used the median of the total scores of worry to categorize it as low worry (< 37) and high worry (≥ 37). We also categorized the scores of four factors of worry using their median scores. We performed binary univariate logistic regression analyses to investigate if there are significant associations between socio-demographic/obstetric variables and the levels of well-being scores as well as the levels of the worry scores. Then, we entered eight variables with *p* < 0.25 into a multivariate logistic regression analysis with backward LR method to identify predictors of the well-being in pregnant women. We also entered fifteen variables with *p* < 0.25 into the multivariate regression analysis to reveal predictors of the worry in pregnant women.

## Results

This descriptive cross-sectional study was conducted on 484 pregnant women participated in the study between May 5 and Aug 5, 2020. Table 1 shows the characteristics of the participating samples. The mean age, gestational age, and years of education were 28.3 ± 5.8 year (range: 16–47), 24.3 ± 8.9 weeks (range: 4–40), and 13.6 ± 3.6 year (range: 2–25), respectively. All women were married. Of the 484 women, 234 (48.3%) were nulliparous, 22.9% were employed, 41.1% had a university degree, and 85.4% were in a middle or high-income category. Among women who were employed, 54 women had been working at home since the COVID-19 outbreak.

**Table 1:** Women’s worry by their socio-demographic/obstetric characteristics (*N* = 484)

		Levels of worry		
		Worry score < 37	Worry score ≥ 37	
*Socio-demographic variables*	N (%)	238 (49.2)	246 (50.8)	P
Age (year)				0.001^†ab^
< 20	28 (5.8)	12 (42.9)	16 (57.1)	
20–30	286 (59.1)	123 (43.0)	163 (57.0)	
> 30	170 (35.1)	103 (60.6)	67 (39.4)	
Job				0.038^†ab^
Housewife	373 (77.1)	193 (51.7)	180 (48.3)	
Employed	111 (22.9)	45 (40.5)	66 (59.5)	
Education level				0.210^†ac^
Primary School	26 (5.4)	16 (61.5)	10 (38.5)	
Diploma	183 (37.8)	95 (51.9)	88 (48.1)	
University	275 (41.1)	127 (46.2)	148 (53.8)	
Family income				0.005^†ab^
Low income	71 (14.7)	23 (32.4)	48 (28.3)	
Middle income	401 (82.9)	207 (51.6)	194 (48.4)	
High income	12 (2.5)	8 (66.7)	4 (33.3)	
Husband’s age				0.016^†ab^
< 30	135 (27.9)	53 (39.3)	82 (60.7)	
30–40	291(60.1)	151 (51.9)	140 (48.1)	
> 40	58 (12)	34 (58.6)	24 (41.4)	
Husband’s education level				0.070^†ab^
Primary School	56 (11.6)	34 (60.7)	22 (39.3)	
Diploma	201 (41.5)	103(51.2)	98 (48.8)	
University	227 (31.8)	101 (44.5)	126 (55.5)	
Husband’s job				0.081^†ab^
Worker	117 (24.2)	47 (40.2)	70 (59.8)	
Clerk	128 (26.4)	66 (51.6)	62 (48.4)	
Self-employed	239 (49.4)	125 (52.3)	114 (47.7)	
Close family member with chronic disease				0.444^a^
Yes	85 (17.6)	45 (52.9)	40 (47.1)	
No	399 (82.4)	193 (48.4)	206 (51.6)	
COVID-19 infected person among relatives				0.009^†ab^
Yes	38 (7.9)	11 (28.9)	27 (71.1)	
No	446 (92.1)	227 (50.9)	219 (49.1)	
Death due to COVID-19 among relatives				0.402^a^
Yes	20 (4.1)	8 (40.0)	12 (60.0)	
No	464 (95.9)	230 (49.6)	234 (50.4)	
Fear of COVID-19				< 0.001^†ab^
Not at all to moderate	272 (70.8)	185 (68.0)	87 (32.0)	
High to Severe	212 (29.2)	53 (25.0)	159 (75.0)	
Participation in the study				0.002^†ab^
After the first wave of COVID-19	384 (79.3)	175 (45.6)	209 (54.4)	
During the second wave of COVID-19	100 (20.7)	63 (63.0)	37 (37.0)	
*Obstetrics variables*				
Gestational age (week)				0.001^†ab^
First trimester	75 (15.5)	51 (68.0)	24 (32.0)	
Second trimester	187 (38.6)	81 (43.3)	106 (56.7)	
Third trimester	222 (45.9)	108 (47.7)	116 (52.3)	
Parity				0.002^†ab^
Nullipara	234 (48.3)	98 (41.9)	136 (58.1)	
Primipara/Multipara	250 (52.7)	140 (56.0)	110 (44.0)	
Having a chronic disease				0.064^†ac^
Yes	15 (3.1)	4 (26.7)	11 (73.3)	
No	469 (96.9)	234 (49.9)	235 (50.1)	
Having a history of abortion				0.085^†ac^
Yes	131 (27.1)	56 (42.7)	75 (57.3)	
No	353 (72.9)	182 (51.6)	171 (48.4)	
Having a pregnancy complication				0.075^†ac^
Yes	44 (9.1)	16 (36.4)	28 (63.6)	
No	440 (90.9)	222 (50.5)	218 (49.5)	
Having a poor obstetric history				0.109^a^
Yes	54 (11.2)	21 (39.9)	33 (61.1)	
No	430 (88.8)	217 (50.5)	213 (49.5)	

^†^selected for multivariate logistic regression analysis

^a^Pearson chi-square, ^b^univariate logistic regression: *p* < 0.05, ^c^univariate logistic regression: *p* < 0.25

Thirty-eight women reported that they had an infected person among their relatives and 20 reported at least one death due to COVID-19 in their extended families. These two groups had a higher level of fear of COVID-19 than their counterparts (*p* = 0.01 and *p* = 0.002, respectively). There was no significant difference in fear of COVID-19 between nulliparous and primi/multiparous women (*p* = 0.313). Women in their second and third trimester of pregnancy had a higher level of fear of COVID-19 than those in their first trimester. The mean scores of the WHO-5 Well-Being Index and the CWS were (64.9 ± 29.0) and (38.5 ± 22.7), respectively. Of the 484 women, 111 (24.4%) had a low level of well-being requiring further evaluation. The Cronbach’s alpha values for the WHO-5 Well-Being Index and for the CWS in the present study are 0.911 and 0.912, respectively.

Table 1 shows women’s worry by their socio-demographic/obstetric characteristics. Women with one of the following attributes had a higher level of worry in comparison with their counterparts: women’s age < 30 years, spouse’s age < 30 years, nulliparous, employed, those with a low family income, those who were in the second and third trimester of pregnancy, those with a high level of fear of COVID-19, and those who had at least one COVID-19 infected person in their relatives (*p* < 0.05). Women who participated in the study during the first wave of the COVID-19 had a higher level of worry than those who registered during the second wave of the disease in Iran (*p* < 0.001).

The results of multivariate logistic regression analysis on worry scores indicates that the predictors of a high level of women’s worry are the increased level of fear of COVID-19 (OR = 6.40, *p* < 0.001), a low family income (OR = 3.41, *p* < 0.001), employment status (OR = 1.86, *p* = 0.019), nulliparity (OR = 1.68, *p* = 0.024), having a COVID-19 infected person among relatives (OR = 2.45, *p* = 0.036), having a history of abortion (OR = 1.86, *p* = 0.012), having participated in the study after the first wave of COVID-19 outbreak (OR = 2.328, *p* = 0.003), and women’s age < 30 (OR = 2.11, *p* = 0.002) (Table 2).

**Table 2:** Results of multivariate logistic regression analysis on worry scores^a^ (*N* = 484)

							95.0% CI for OR	
Model	B	S.E.	Wald	df	P	OR	Lower Bound	Upper Bound
Age								
> 30						1		
< 20	.881	.504	3.061	1	0.080	2.414	.899	6.481
20–30	.749	.245	9.329	1	0.002	2.114	1.308	3.417
Job								
Housewife						1		
Employed	.618	.264	5.493	1	0.019	1.855	1.106	3.110
Having a COVID-19 infected person among relatives								
No						1		
Yes	.895	.428	4.375	1	0.036	2.448	1.058	5.664
Fear of COVID-19								
Low fear						1		
High fear	1.856	.221	70.272	1	< 0.001	6.397	4.145	9.873
Parity								
Primi/multiparity						1		
Nulliparity	.517	.229	5.115	1	0.024	1.678	1.071	2.627
Having a history of abortion								
No						1		
Yes	.622	.247	6.346	1	0.012	1.863	1.148	3.022
Participation in the study								
During the second wave of COVID-19 outbreak						1		
After the first wave of COVID-19 outbreak	.845	.286	8.741	1	0.003	2.328	1.329	4.075
Family income								
High income						1		
Low/middle income	1.227	.323	14.455	1	< 0.001	3.411	1.812	6.421

^a^low level of worry (scores < 37), high level of worry (scores ≥37)

Fifteen variables with *p* < 0.25 were entered into the regression; eight variables remained in the model

Cox & Snell R Square = 25.7%, Nagelkerke R Square = 0.343

Table 3 shows women’s well-being by their socio-demographic/obstetric characteristics. Women with one of the following attributes had a lower level of well-being in comparison with their counterparts: women with at least one COVID-infected person among relatives, those with at least one death among their relatives due to COVID-19, and women with a high level of fear of COVID-19 (*p* < 0.05). Table 4 indicates that there are statistically significant differences in the mean scores of the Cambridge worry factors for subgroups of women classified according to their well-being score.

**Table 3:** Women’s well-being by their socio-demographic characteristics (*N* = 484)

		Levels of well-being		
		WHO-5 index < 50	WHO-5 index ≥50	
*Socio-demographic variables*	N (%)	118 (24.4)	366 (75.6)	P
Age (year)				0.847^a^
< 20	28 (5.8)	8 (28.6)	20 (71.4)	
20–30	286 (59.1)	68 (23.8)	218 (76.2)	
> 30	170 (35.1)	42 (24.7)	128 (75.3)	
Job				0.441^a^
Housewife	373 (77.1)	94 (25.2)	279 (74.8)	
Employed	111 (22.9)	24 (21.6)	87 (78.4)	
Education				0.060^a^
Primary School	26 (5.4)	4 (15.4)	22 (84.6)	
Diploma	183 (37.8)	55 (30.1)	128 (69.9)	
University	275 (41.1)	59 (21.5)	216 (78.5)	
Family income				0.232^ac^
Low	71 (14.7)	23 (32.4)	48 (77.6)	
Middle	401 (82.9)	92 (22.9)	309 (77.1)	
High	12 (2.5)	3 (257)	9 (75)	
Husband’s age				0.313^a^
< 30	135 (27.9)	28 (20.7)	107 (79.3)	
30–40	291(60.1)	78 (26.8)	213 (73.2)	
> 40	58 (12)	12 (20.7)	46 (79.3)	
Husband’s education level				0.217^a^
Primary School	56 (11.6)	13 (23.2)	43 (76.8)	
Diploma	201 (41.5)	57 (28.4)	144 (71.6)	
University	227 (31.8)	48 (21.1)	179 (78.9)	
Husband’s job				0.167^a^
Worker	117 (24.2)	35 (29.9)	82 (70.1)	
Clerk	128 (26.4)	25 (19.5)	103 (80.5)	
Self-employed	239 (49.4)	58 (24.3)	181 (75.7)	
Close family member with chronic disease				0.300^a^
Yes	85 (17.6)	17 (20.0)	68 (80.0)	
No	399 (82.4)	101 (25.3)	298 (74.7)	
COVID-19 infected person among relatives				0.008^†ab^
Yes	38 (7.9)	16 (42.1)	22 (57.9)	
No	446 (92.1)	102 (22.9)	344 (77.1)	
Death due to COVID-19 among relatives				0.032^‡ab^
Yes	20 (4.1)	9 (45.0)	11 (55.0)	
No	464 (95.9)	109 (23.5)	355 (76.5)	
Fear of COVID-19				0.016^ab^
Not at all to moderate	272 (70.8)	55 (20.2)	217 (79.8)	
High to Severe	212 (29.2)	63 (29.7)	149 (70.3)	
Participation in the study				0.377^a^
After the first wave of COVID-19	384 (79.3)	97 (25.3)	287 (74.7)	
During the second wave of COVID-19	100 (20.7)	21 (21.0)	79 (79.0)	
*Obstetrics variables*				
Gestational age (week)				0.513^a^
First trimester	75 (15.5)	21 (28.0)	54 (72.0)	
Second trimester	187 (38.6)	48 (25.7)	139 (74.3)	
Third trimester	222 (45.9)	49 (22.1)	173 (77.9)	
Parity				0.992^a^
Nullipara	234 (48.3)	57 (24.4)	177 (75.6)	
Primipara/Multipara	250 (52.7)	61 (24.4)	189 (75.6)	
Having a history of abortion				0.823^a^
Yes	131 (27.1)	31 (23.7)	100 (76.3)	
No	353 (72.9)	87 (24.6)	266 (75.4)	
Having a pregnancy complication				0.920^a^
Yes	44 (9.1)	11 (25)	33 (75)	
No	440 (90.9)	107 (24.3)	333 (75.7)	
Having a poor obstetric history				0.341^a^
Yes	54 (11.2)	16 (29.6)	38 (70.4)	
No	430 (88.8)	102 (23.7)	328 (76.3)	

^a^Pearson chi-square, ‡Fisher exact test, ^b^logistic regression: *p* < 0.05, ^c^logistic regression: *p* < 0.25

†selected for multivariate logistic regression analysis

**Table 4:** Differences in the sub-groups of worry by level of well-being (*N* = 484)

		Levels of well-being		p
Worry factors		WHO-5 < 50 (*n* = 118)	WHO-5 ≥ 50 (*n* = 366)	
Socio Medical	19.9 ± 12.2	7.2 ± 5.8	5.5 ± 5.3	0.002^ab^
Health of mother & Relationships	5.6 ± 4.7	7.3 ± 4.8	5.1 ± 4.5	0.001^ab^
Fetus Health	6.5 ± 4.8	8.3 ± 4.7	5.9 ± 4.6	< 0.001^ab^
Socio Economic	5.9 ± 5.5	23.5 ± 11.7	18.7 ± 12.1	0.091^ac^

^a^Man-Whitney U

^b^Logistic regression: *p* < 0.05

^c^Logistic regression: *p* = 0.055

The results of the multivariate logistic regression analysis on well-being scores showed that the predictors of low level of well-being in pregnant women are worry about their own health and relationships (OR = 1.789, *p* = .017), worry about fetus health (OR = 1.946, *p* = 0.009), and having at least one infected person with COVID-19 among relatives (OR = 2.135, *p* = 0.036) (Table 5).

**Table 5:** Results of multivariate logistic regression analysis on well-being scores (scores ≥50 vs. scores < 50)^a^

							95% C.I. For OR	
Variables	B	S.E.	Wald	df	P	OR	Lower	Upper
Having an infected person with COVID-19 among relatives								
No						1		
Yes	0.786	0.356	4.880	1	0.027	2.194	1.093	4.405
Worry about health of mother & relationships^b^								
Low worry (scores < 5.6)						1		
High worry (scores ≥5.6)	0.528	0.239	4.867	1	0.027	1.696	1.061	2.712
Worry about fetus health^b^								
Low worry (scores < 6.5)						1		
High worry (scores ≥6.5)	0.625	0.251	6.181	1	0.013	1.868	1.141	3.057

^a^low level of well-being (scores < 50), high level of well-being (scores ≥50), ^b^the median score as cut-off point

Eight variables entered the logistic regression analysis as independent variable include infection with COVID-19 in relatives, death due to COVID-19 in relatives, fear of COVID, family income, four factors of the worry scale. Method of analysis: Backward LR, Cox & Snell R Square = 0.052, Nagelkerke R Square = 0.078

## Discussion

We investigated well-being and worry and associated factors with each one in pregnant women during the COVID-19 pandemic. Our findings show that predictors of women’s worry are the increased level of fear of COVID-19, nulliparity, employment status, a low family income, women’s age < 30, having a history of abortion, having a COVID-19 infected person among relatives, and having participated in the study after the first wave of the COVID-19 outbreak.

We found that nulliparity is a predictor of women’s worry during COVID-19 pandemic. First time pregnant women had a difficult period both because of the threat of the disease and pregnancy discomforts and complications. Lebel and colleagues also reported a higher level of pregnancy-related anxiety among nulliparous women compared to primi/multiparas during COVID-19 pandemic [3]. In contrast, Effati-Daryani and colleagues reported a lower level of anxiety in nulli/primiparous women than multiparas in a study on 205 pregnant women during the outbreak of COVID-19 in Iran [18]. In our study, pregnant women younger than 30 years had a higher level of worry than older women. Although parity and maternal age are correlated, both variables remained in the model as predictors of women’s worry.

A finding of our study was that being employed is a predictor of women’s worry. This may be due the fact that women perceived that compared to the risk faced by homemakers, there was a higher risk of being infected in the work environment during COVID-19 pandemic. Effati-Daryani and colleagues reported a higher level of stress among pregnant women whose spouse was a shopkeeper compared to those whose spouse was a clerk [18]. Our results show that insufficient family income is a predictor of women’s worry. Although this result seems reasonable, Effati-Daryani and colleagues reported a reverse association so that women with fairly to completely sufficient income had a higher level of stress in comparison with those who had insufficient income [18]. In a study on 2740 pregnant women from 47 countries, more than half of the women reported increased stress about household income during the COVID-19 pandemic [19].

We found that having a history of abortion is a predictor of women’s worry during COVID-19 outbreak. Pregnant women who had a previous abortion were worried about a repeat abortion. Previous research indicates that women with a history of abortion are prone to elevated rates of mental illness compared to women with no such history [20]. Such concerns about a repeat abortion must have been exacerbated during COVID-19 outbreak because of the possibility of harm to the fetus by the virus. In our study, having a COVID-19 infected person among relatives is a predictor of women’s worry. This would usually increase the perceived risk of contracting the disease. In a multinational study on pregnant women, 93% of women reported increased levels of stress about getting infected with COVID-19 [19]. In a study at the start of COVID-19 pandemic on 439 individuals, perceived risk of loved ones contracting coronavirus was a predictor of fear of COVID-19 [21].

Our results indicate that the level of worry was lower among pregnant women who participated in the study during the second wave of COVID-19 epidemic compared to those who participated in the study during the first wave of the disease. Further studies are needed to investigate the effects of COVID-19 pandemic’s successive phases on individuals’ level of worry and mental health.

Results indicate that predictors of low level of well-being in pregnant women are worry about their own health and relationships, concerns about fetus health, and having at least one infected person with COVID-19 among relatives. Pregnant women are concerned about the health of the fetus in addition to their own health. Even before the COVID-19 outbreak, the possibility of giving birth to an unhealthy baby was the most prevalent causes of worry and anxiety in pregnant women [12, 22]. Lebel and colleagues reported similar results in a study on 1987 pregnant women. They found that pregnant women who were worried about the health of their baby and their own health and their relationships with close family during the COVID-19 pandemic showed higher symptoms of depression and anxiety [3].

Having an infected person with COVID-19 among relatives is a predictor of women’s level of worry. It is also a predictor of low level of well-being. In a study on 253 Italian postpartum women, women who worried about a close acquaintance being infected and those with a close one actually infected showed higher symptoms of depression than their counterparts [23].

We found that the percentage of women experiencing a low well-being state was 24.4% which is relatively high in comparison with previous studies. These percentages were 9.18% in Mumbai [24] and 19.6% in Osasco, São Paulo [25]. The percentage of participants with a low level of well-being in our previous study conducted before the pandemic was 25.2% [26], a proportion which did not significantly differ from that of the present study. This result is not in agreement with a previous Canadian study carried on 1987 pregnant women conducted on 5–20 March 2020. The Canadian study found an elevated rate of depression and anxiety symptoms among pregnant women in comparison with previous meta analyses [3]. In another study of 5866 pregnant and breastfeeding women during the lockdown period in Belgium, an elevated rate of depression and anxiety symptoms was reported in comparison with reported rates prior to the pandemic [27]. In fact, most studies were conducted in the COVID-19 pandemic early on or in the quarantine period when psychological well-being of pregnant women had been severely affected. We conducted this study in the period after the first wave of the COVID-19 outbreak in Iran when the quarantine period had ended and COVID-19 infection rates and death toll had decreased. These favorable developments might have been interpreted by women as a sign that the pandemic was coming to an end. That may have affected positively pregnant women’s feelings and functioning.

In comparison with our previous study before the pandemic, women’s concerns about fetus health had decreased. In our previous study, the mean score of fetus health factor was 7.2 ± 4.7 [12]. The availability of ultrasound scans during recent years may have had a role in decreasing the women’s worries about fetus health. The results of a study on postpartum women indicates that on average women receive 5.9 ultrasound scans during pregnancy. Obtaining assurance about fetus health was the first reason given by women for undergoing ultrasound scans [28]. In addition, in 2014, the health transformation plan (HTP) was launched in the Iranian health system. One of the HTP packages had been designed with specific purpose of improving maternal health [29]. The package included several interventions such as holding free childbirth preparatory classes in both hospitals and health centers and providing the option of having a midwife at birth for pregnant women. Women participating in this study had already taken part in the virtual childbirth preparatory classes. They also had the option of having a midwife at labor. It is possible that these interventions had reduced concerns about fetus health among pregnant women. Lebel and colleagues also reported lower levels of anxiety and depression symptoms among pregnant women who received general support compared with those who did not [3].

In comparison with our previous study before the pandemic, women’s worries about socio economic matters had increased. In our previous study, the mean score of socio-economic factor was 5.2 ± 4.5 [12]. COVID-19 pandemic has negatively affected the economy almost in all countries [30] leading to increased poverty rate [31]. Therefore, it is not surprising to find that women were experiencing higher levels of worry about household livelihood and expenses compared to the prepandemic period. To decrease women’s socio economic concerns, governments and charities should expand programs providing financial support to pregnant women in low-income families.

The first limitation of our study was that because it was an online survey, women who did not have a smartphone were not able to participate in it. It is likely that these women belonged to a lower socio-economic class than those who participated in the study. Also, the economic hardships that they may have faced would have influenced their level of worries. The second limitation of the study is that women who were more concerned and worried were more likely not to participate in the study than those who were less worried. These two limitations may have led to a lower observed level of worry and higher observed well-being among the women in the present study compared to our previous studies. The sample in the present study consists of women with higher education levels than the study conducted in 2019 [26]. This means that the proportion of women with higher education is higher among the participants in our study compared to the previous study. We believe that these women had a lower level of worry than women with lower levels education because they had access to multiple sources of news and information and were able to evaluate COVID-19 news. It is because of these factors that we believe the reported level of well-being in women in the present study might be higher than average. This might explain why we found no difference between the levels of well-being between the present study and the previous study.

Online surveys have their own strengths and weaknesses. They save time for both participants and investigators. It is possible to design the questionnaire in such a way that participants do not miss any questions or items. Another strength point of this study is that we could perform comparisons between this study, which was conducted during the COVID-19 period and two previous studies which were published in 2019 and 2016. The third strong point of the study is that we used the same scales to measure well-being and worry in all the three studies mentioned above. This enabled us to compare worry and well-being between the studies. This post-quarantine study provided us with an opportunity to examine the impact of this prolonged pandemic on women’s well-being and worry.

## Conclusions

In this study, we investigated worry and well-being in pregnant women during the COVID-19 pandemic. To summarize, our findings call attention to the problems of pregnant women who have one or more of the following characteristics: nulliparous, being in paid employment, low-income, having a history of abortion, having a COVID-19 infected person among relatives, women’s age < 30 and being severely fearful of COVID-19. We recommend setting up support groups for these women to help them overcome their worries during the pandemic.

Our results indicate a close relationship between worry and well-being in pregnant women. More precisely, pregnant women’s worries about the health of their fetus, their own health and about their relationships with their husbands and family members had negative impact on their well-being. These findings call for actions by health care providers and in particular midwives with the aim of supporting pregnant women. In the current circumstances, this can be achieved best by setting up online groups to attend to their concerns and refer those with high levels of worry to counselors.

We also found that the percentage of women experiencing a low well-being state was relatively high, a result worthy of attention by health care providers and policy makers. Providing care and support to pregnant women, particularly the more disadvantaged and vulnerable groups should have high priority during the COVID-19 pandemic. Public health authorities should plan for situations like this in advance and should be prepared to adopt appropriate measures to reduce pregnant women’s concerns.

## Supplementary Information

**Additional file 1.**

## Data Availability

The data that support the findings of this study are available from the corresponding author upon a reasonable request.

## Abbreviations

WHO-5 well-being index: World Health Organization’s well-being Index
CWS: Cambridge worry scale
COVID-19: Corona Virus Disease-2019
