# Supplementary material for: Pregnant women’s well-being and worry during the COVID-19 pandemic: a cross-sectional study
Source: BMC Pregnancy Childbirth. 2021 Jan 15;21:59. doi: 10.1186/s12884-021-03548-4 (PMC7809640; doi:10.1186/s12884-021-03548-4)
Supplement: Supplementary file 1 — Additional file 1. [file 12884_2021_3548_MOESM1_ESM.docx]

Dear Respondent.

We are going to investigate pregnant women’s well-being and worries during COVID-19 epidemic. We appreciate your assistance in this project.

**Please answer the questions carefully.**

What is your gestational age based on your health records? ..…. week

How old are you? …… year

How many years have you studied? …… year

What is your job? 1. Homemaker 2. Employed

Had you been working at home since the COVID-19 outbreak? 1. Yes 2. No

How many times have you been pregnant? ………….

How many times have you given birth? ..…………

Do you have a history of miscarriage? 1. Yes 2. No

Do you have a chronic disease? 1. Yes 2. No

Have you had any severe pregnancy complication such as gestational diabetes, hypertension, hemorrhage, and so on during this pregnancy? 1. Yes 2. No

Do you have a poor obstetric history such as infertility, habitual abortion, infant or fetal death, and so on? 1. Yes 2. No

What is your family income? 1. Low 2. Middle 3. High

How old is your husband? ………… year

What is your husband job?

1. Worker 2. Clerk 3. Self-employed 4. Teacher 5. Others

How many years have your spouse studied? …………… year

Does your spouse have a chronic illness? 1. Yes 2. No

Does one of your close family member have a chronic disease? 1. Yes 2. No

How scared are you of COVID-19?

1. Not at all 2. Low 3. Moderate 4. High 5. Severe

Have you had at least one COVID-19 infected person among relatives? 1. Yes 2. No

Have you had at least one death due to COVID-19 among relatives? 1. Yes 2. No

WHO-5 well-being Index

Please respond to each item by marking one box per row, regarding how you felt in the last two weeks

|  | All of the time | Most of the time | More than half the time | Some of the time | Less than half the time | At no time |
| --- | --- | --- | --- | --- | --- | --- |
| I have felt cheerful in good spirits | 5 | 4 | 3 | 2 | 1 | 0 |
| I have felt calm and relaxed | 5 | 4 | 3 | 2 | 1 | 0 |
| I have felt active and vigorous | 5 | 4 | 3 | 2 | 1 | 0 |
| I woke up feeling fresh and rested | 5 | 4 | 3 | 2 | 1 | 0 |
| My daily life has been filled with things that interest me. | 5 | 4 | 3 | 2 | 1 | 0 |

**The Persian Worry scale**

|  | Not a worry | 1 | 2 | 3 | 4 | Major worry |
| --- | --- | --- | --- | --- | --- | --- |
|  | 0 | 1 | 2 | 3 | 4 | 5 |
| Having nobody in delivery ward | 0 | 1 | 2 | 3 | 4 | 5 |
| Giving birth | 0 | 1 | 2 | 3 | 4 | 5 |
| Possibility of fetal death, disease or anomaly | 0 | 1 | 2 | 3 | 4 | 5 |
| Whether midwives provide good care in labor | 0 | 1 | 2 | 3 | 4 | 5 |
| Internal examinations | 0 | 1 | 2 | 3 | 4 | 5 |
| Whether your husband will be with you at the time of admission to labor | 0 | 1 | 2 | 3 | 4 | 5 |
| Crowded delivery ward | 0 | 1 | 2 | 3 | 4 | 5 |
| Probability of not having a spontaneous labor | 0 | 1 | 2 | 3 | 4 | 5 |
| Possibility of miscarriage | 0 | 1 | 2 | 3 | 4 | 5 |
| Probability of going into labor too early | 0 | 1 | 2 | 3 | 4 | 5 |
| Going to hospital | 0 | 1 | 2 | 3 | 4 | 5 |
| Money problems | 0 | 1 | 2 | 3 | 4 | 5 |
| Coping with the new baby | 0 | 1 | 2 | 3 | 4 | 5 |
| Your own health | 0 | 1 | 2 | 3 | 4 | 5 |
| Health of relatives | 0 | 1 | 2 | 3 | 4 | 5 |
| Employment problems | 0 | 1 | 2 | 3 | 4 | 5 |
| Relationship with husband | 0 | 1 | 2 | 3 | 4 | 5 |
| Your housing | 0 | 1 | 2 | 3 | 4 | 5 |
| Unplanned or unwanted pregnancy | 0 | 1 | 2 | 3 | 4 | 5 |
| Relationship with family | 0 | 1 | 2 | 3 | 4 | 5 |
| Problems with the law | 0 | 1 | 2 | 3 | 4 | 5 |
| Baby gender | 0 | 1 | 2 | 3 | 4 | 5 |
